# A genome-wide association scan for rheumatoid arthritis data by Hotelling's *T*^2 ^tests

**DOI:** 10.1186/1753-6561-3-s7-s6

**Published:** 2009-12-15

**Authors:** Lianfu Chen, Ming Zhong, Wei Vivien Chen, Christopher I Amos, Ruzong Fan

**Affiliations:** 1 Department of Statistics, Texas A&M University, 447 Blocker Building, College Station, TX 77843, USA; 2 Department of Epidemiology, MD Anderson Cancer Center, University of Texas, 1515 Holcombe Boulevard, Houston, TX 77030, USA

## Abstract

We performed a genome-wide association scan on the North American Rheumatoid Arthritis Consortium (NARAC) data using Hotelling's *T*^2 ^tests, i.e., *T*_*H *_based on allele coding and *T*_*G *_based on genotype coding. The objective was to identify associations between single-nucleotide polymorphisms (SNPs) or markers and rheumatoid arthritis. In specific candidate gene regions, we evaluated the performance of Hotelling's *T*^2 ^tests. Then Hotelling's *T*^2 ^tests were used as a tool to identify new regions that contain SNPs showing strong associations with disease. As expected, the strongest association evidence was found in the region of the HLA-DRB1 locus on chromosome 6. In the region of the TRAF1-C5 genes, we identified two SNPs, rs2900180 and rs3761847, with the largest and the second largest *T*_*H *_and *T*_*G *_scores among all SNPs on chromosome 9. We also identified one SNP, rs2476601, in the region of the PTPN22 gene that had the largest *T*_*H *_score and the second largest *T*_*G *_score among all SNPs on chromosome 1. In addition, SNPs with the largest *T*_*H *_score on each chromosome were identified. These SNPs may be located in the regions of genes that have modest effects on rheumatoid arthritis. These regions deserve further investigation.

## Background

Rheumatoid arthritis (RA) is the most common inflammatory joint disease and has an autoimmune etiology. The exact cause of RA is still unknown, but it is well known that RA has a strong genetic component [1]. The HLA-DRB1 locus has been clearly demonstrated to be associated with RA [2-4]. Other candidate genes, such as PTPN22 and TRAF1-C5, which confer a modest level of risk of RA, have also been identified recently [5,6]. We conducted a genome-wide association analysis on the data of the North American Rheumatoid Arthritis Consortium (NARAC). The objective of this analysis was to identify associations between single-nucleotide polymorphisms (SNPs) or markers and RA. In specific candidate gene regions, we evaluated the performance of Hotelling's *T*^2 ^tests on known associations. Then, we used the Hotelling's *T*^2 ^tests to identify additional SNPs that showed strong association with RA. These SNPs are located in regions that are very likely related to the disease and deserve further investigation.

## Methods

We used the Hotelling's *T*^2 ^test developed by Fan and Knapp [7] and Xiong et al. [8] to analyze the NARAC data. Consider a case-control design with *N *cases from an affected population and *M *controls from an unaffected population. When analyzing SNPs, we study bi-allelic markers with two alleles, which we denoted by 1 and 2 that can form three genotypes 1/1, 1/2 and 2/2. Then a coding vector can be defined for each case/control by either i) genotype coding or ii) allele coding. Let *X*_*i *_and *Y*_*j *_denote the coding vector for the *i*^th ^case and the *j*^th ^control, respectively. In our study, *X*_*i *_= (1,0)^τ^ for genotype 1/1, *X*_*i *_= (1,0)^τ^ for genotype 1/2, and *X*_*i *_= (0,0)^τ^ for genotype 2/2 were used in the genotype coding, whereas the allele coding simply counts the number of allele 1 of a genotype. If multiple markers are available, the coding vectors of each case/control can be combined together. For instance, the allele coding vector of a case/control of *n *SNPs is an *n*-dimensional vector; and the genotype coding vector of a case/control of *n *SNPs is 2*n*-dimensional. For multi-allelic markers, the coding method is described by Fan and Knapp [7]. Let us define a pooled-sample variance covariance matrix by

where  and  are the mean vectors of cases and controls, respectively. The Hotelling's *T*^2 ^test statistic [9] is defined as

In the following, we will denote the Hotelling's *T*^2 ^for allele coding as *T*_*H *_and the Hotelling's *T*^2 ^for genotype coding as *T*_*G*_. Assume the sample sizes *N *and *M *are large enough so that the large sample theory applies. Under the null hypothesis of no association, the statistic *T*_*H *_(or *T*_*G*_) is asymptotically distributed as a central chi-square *χ*^2 ^statistic with *n *(or *2n*) degree(s) of freedom if *n *SNPs are used in the analysis. Under the alternative hypothesis of association, *T*_*H *_(or *T*_*G*_) is asymptotically distributed as a non-central chi-square *χ*^2 ^statistic [7,8,10].

Based on the Hotelling's *T*^2 ^test statistics, we have developed a SAS Macro (hotel_cc.sas) to implement the method, which is available online [11].

## Results

**Figure 1:**
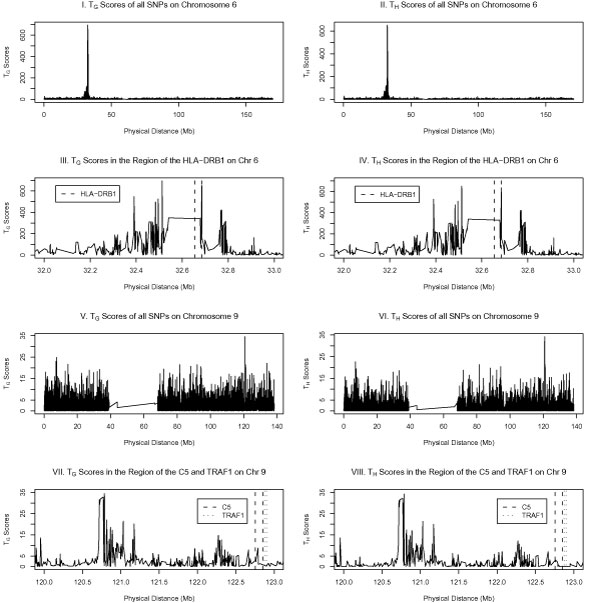
**Hotelling's test scores for chromosomes 6 and 9 data**.

First, we applied the Hotelling's test statistics and performed a genome-wide scan on the NARAC data by analyzing one SNP at a time. The NARAC data contained a total of 2062 individuals (868 cases and 1194 controls). Our analysis used data from 22 autosomes. The RA data of Genetic Analysis Workshop (GAW) 16 included 545,080 SNP-genotype fields from an Illumina 550 k chip (22 autosomes, sex chromosomes, and mitochondria). We dropped all SNPs with low call rates (less than 95%) or not in Hardy-Weinberg equilibrium in the controls (*p*-value < 10^-5^) and dropped all SNPs which are not on the autosomes. After this filtering, 490,613 SNPs on 22 autosomes were used in our analysis. The strongest signal was found in the region of the HLA-DRB1 gene on chromosome 6 at location 32,654,524-32,686,031 bp. In Figure 1, Graphs I and II show the Hotelling's test scores for chromosome 6. Both *T*_*H *_and *T*_*G *_scores reached the highest value around the location of 32.5 Mb in the region of HLA-DRB1. Graphs III and IV showed the results in the region of HLA-DRB1 gene (the legend indicates location of the HLA-DRB1 gene). Most of the test scores in the region were very significant.

We present the six SNPs on chromosome 6 with the highest test scores in the left-hand part of Table 1. The most significant result was found at SNP rs2395175 (*p*-value = 9.25 × 10^-144^). These SNPs are all located around the HLA-DRB1 gene. It is interesting that both *T*_*H *_and *T*_*G *_reached the highest scores at the same four SNPs (rs2395175, rs660895, rs6910071, and rs2395163). Interstingly, *T*_*H *_reached the 5^th ^highest score at SNP rs3763309 and the 6^th ^highest at SNP rs3763312; conversely, *T*_*G *_reached the 5^th ^highest score at SNP rs3763312 and the 6^th ^highest at SNP rs3763309. Actually, the order of two SNPs for *T*_*H *_and *T*_*G *_that reached the 7^th ^and 8^th ^highest scores switches too; in addition, *T*_*H *_and *T*_*G *_reached the 9^th ^to 13^th ^highest scores at the same SNPs (data not shown). Thus, the region of the HLA-DRB1 gene contains multiple SNPs that are highly associated with RA. In addition, the *p*-values of the test *T*_*G *_were generally smaller than those of *T*_*H*_, i.e., the genotype coding test *T*_*G *_leads to more significant results than the allele coding test *T*_*H*_. This observation is consistent with the evidence for non-additivity of DRB1 effects [12].

**Table 1:** Six SNPs on chromosome 6 and 9 with the highest test scores

Highest Test Scores of Chromosome 6				Highest Test Scores of Chromosome 9			
	
SNP	Position (bp)	Test score	*p*-Value	SNP	Position (bp)	Test score	*p*-Value
rs2395175	32513004	*T*_*H *_= 651.76	9.25 × 10^-144^	rs2900180	120785936	*T*_*H *_= 34.21	4.95 × 10^-9^
rs660895	32685358	*T*_*H*_= 635.76	2.79 × 10^-140^	rs3761847	120769793	*T*_*H *_= 32.17	1.41 × 10^-8^
rs6910071	32390832	*T*_*H *_= 527.23	1.13 × 10^-116^	rs881375	120732452	*T*_*H *_= 31.64	1.86 × 10^-8^
rs2395163	32495787	*T*_*H *_= 509.44	8.4 × 10^-113^	rs1953126	120720054	*T*_*H *_= 31.19	2.34 × 10^-8^
rs3763309	32483951	*T*_*H *_= 474.63	3.15 × 10^-105^	rs10760130	120781544	*T*_*H *_= 30.1	4.10 × 10^-8^
rs3763312	32484326	*T*_*H*_= 472.10	1.12 × 10^-104^	rs10985073	120723409	*T*_*H *_= 28.16	1.12 × 10^-7^

rs2395175	32513004	*T*_*G *_= 694.14	1.87 × 10^-151^	rs2900180	120785936	*T*_*G *_= 34.51	3.21 × 10^-8^
rs660895	32685358	*T*_*G *_= 653.09	1.53 × 10^-142^	rs3761847	120769793	*T*_*G *_= 32.82	7.48 × 10^-8^
rs6910071	32390832	*T*_*G *_= 548.25	8.89 × 10^-120^	rs881375	120732452	*T*_*G *_= 31.88	1.19 × 10^-7^
rs2395163	32495787	*T*_*G *_= 526.00	6.03 × 10^-115^	rs1953126	120720054	*T*_*G *_= 31.69	1.32 × 10^-7^
rs3763312	32484326	*T*_*G *_= 496.90	1.26 × 10^-108^	rs10760130	120781544	*T*_*G *_= 31.27	1.62 × 10^-7^
rs3763309	32483951	*T*_*G *_= 495.07	3.14 × 10^-108^	rs10985073	120723409	*T*_*G *_= 29.18	4.62 × 10^-7^

It is well known that the HLA-DRB1 alleles are associated with RA [1,2]. We performed an analysis in which HLA-DRB1 alleles *0101, *0102, *0401, *0404, *0405, *0408, *1001, which are components of the shared epitope were treated as risk alleles, and the other alleles were collapsed as one. Here we used the multi-allelic version of the Hotelling's *T*^2 ^tests [7]. The test score for allele coding was *T*_*H *_= 650.81 with 7 degrees of freedom (*p*-value = 2.76 × 10^-136^), and test score for genotype coding was *T*_*G *_= 694.82 with 35 degrees of freedom (*p*-value = 1.36 × 10^-123^). The results were consistent with those using individual SNPs above. On the basis of individual SNP analysis, we performed a forward analysis of multiple SNPs. Using the most significant SNP rs2395175 as baseline, we added one SNP a time for an analysis of two SNPs. We identified that each of three SNPs, rs660895, rs6910071, and rs3763312, contributed significant association in addition to the contribution of the base SNP rs2395175 (*p*-value < 0.01). Moreover, the most significant result was from the two SNPs rs2395175 and rs660895. Then, we added one SNP at a time to the two most significant SNPs; we found each of the two SNPs, rs6910071 and rs3763312, contributed significant association (*p*-value < 0.01). Finally, four SNPs together were found to be significantly associated with RA (rs2395175, rs660895, rs6910071 and rs3763312; *p*-value < 0.01).

Graphs V-VIII of Figure 1 showed the results of chromosome 9 (the legend indicates location of theTRAF1-C5 genes). In Plenge et al. [6], SNP rs3761847 at position 120,769,793 bp and SNP rs2900180 at position 120,785,936 bp were found to be significantly associated with RA in the region of the TRAF1-C5 genes. We found consistent results since *T*_*H *_= 34.21 of SNP rs2900180 was the largest (*p*-value = 4.95 × 10^-9^), and *T*_*H *_= 32.17 of SNP rs3761847 was the second largest among all SNPs on chromosome 9 (*p*-value = 1.41 × 10^-8^). Other SNPs on chromosome 9 that showed highest scores were also reported on the right-hand side of Table 1. Interestingly, the SNPs identified via *T*_*H *_were the same as the ones identified via *T*_*G *_(the right-hand side of Table 1). As with chromosome 6 in the region HLA-DRB1, we performed a forward analysis of multiple SNPs. Using rs2900180 as baseline, we found no other SNP that contributed significant association (*p*-value > 0.05). Thus, all association is from SNP rs2900180 in the region of the TRAF1-C5 genes.

In the region of the PTPN22 gene on chromosome 1, we identified one SNP (rs2476601) that was reported to be associated with RA by Begovich et al. [5]. The SNP is located at position 114,089,610 bp on the left-hand side of the PTPN22 gene. The *T*_*H *_= 48.88 of rs2476601 was the largest *T*_*H *_score among all SNPs on chromosome 1 (*p*-value = 2.72 × 10^-12^), and the *T*_*G *_= 49.99 of rs2476601 was the second largest (*p*-value = 1.4 × 10^-11^, data not shown). In this region, only SNP rs2476601 stood out; other SNPs of top 20 test scores are not located in the region. Hence, we did not analyze multiple SNPs.

From the results in the candidate regions on chromosomes 6, 9, and 1, we noticed that the highest test scores of *T*_*H *_and *T*_*G *_were from SNPs located very close to the candidate genes HLA-DRB1, TRAF1-C5, and PTPN22, respectively. Therefore, the SNPs with high test scores are of interest for further investigation to identify genes that have modest effect on RA. In Table 2, we presented the SNPs that showed the highest *T*_*H *_scores among all SNPs of each chromosome. We chose to present the results based on the test statistic *T*_*H*_, since it is more robust than *T*_*G *_in terms of more stable type I error rates [7]. To make a comparison, we presented the most significant results from PLINK in Table 2. The SNPs identified by statistic *T*_*H *_are the same as those identified by PLINK, except rank switches on chromosomes 11 and 16. It is possible that other SNPs that have high test scores are worthy of further study. Due to the limited length of this article, we could not present detailed genome-wide test data here but we will provide detailed information on request.

**Table 2:** SNPs and positions of the highest *T*_*H *_scores on each chromosome

Chr	SNP	Position	T_H _(*p*-value)	χ^2 ^(*p*-value)
1	rs2476601	114089610	48.88 (2.72 × 10^-12^)	50.62 (1.12 × 10^-12^)
2	rs6433309	172343658	23.37 (1.34 × 10^-6^)	22.88 (1.72 × 10^-6^)
3	rs9290452	174045236	25.01 (5.70 × 10^-7^)	25.04 (5.62 × 10^-7^)
4	rs1388021	157757048	29.12 (6.81 × 10^-8^)	28.97 (7.35 × 10^-8^)
5	rs6596147	133075674	35.82 (2.16 × 10^-9^)	36.21 (1.77 × 10^-9^)
6	rs2395175	32513004	651.76 (9.25 × 10^-144^)	534.10 (3.62 × 10^-118^)
7	rs6978820	146629802	23.69 (1.13 × 10^-6^)	23.54 (1.22 × 10^-6^)
8	rs9785133	20402898	29.44 (5.78 × 10^-8^)	30.20 (3.90 × 10^-8^)
9	rs2900180	120785936	34.21 (4.94 × 10^-9^)	33.76 (6.23 × 10^-9^)
10	rs2671692	49767825	30.61 (3.16 × 10^-8^)	30.94 (2.66 × 10^-8^)
11	rs16935797	8077876	20.72 (5.32 × 10^-6^)	20.80 (5.10 × 10^-6^)
11	rs376813	8221633	19.1 (1.24 × 10^-5^)	21.33 (3.87 × 10^-6^)
12	rs1022232	53231332	23.01 (1.61 × 10^-6^)	22.81 (1.79 × 10^-6^)
13	rs1177637	41590823	20.28 (6.71 × 10^-6^)	19.96 (7.91 × 10^-6^)
14	rs12885166	92195035	25.38 (4.72 × 10^-7^)	25.53 (4.36 × 10^-7^)
15	rs12913832	26039213	22.11 (2.58 × 10^-6^)	23.27 (1.41 × 10^-6^)
16	rs1875206	9966508	23.92 (1.01 × 10^-6^)	23.81 (1.06 × 10^-6^)
16	rs2521669	26795854	23.64 (1.16 × 10^-6^)	24.08 (9.24 × 10^-7^)
17	rs9896052	70930457	23.00 (1.62 × 10^-6^)	23.53 (1.23 × 10^-6^)
18	rs12455894	26873793	26.23 (3.03 × 10^-7^)	25.61 (4.18 × 10^-7^)
19	rs8104309	36858686	26.89 (2.16 × 10^-7^)	26.15 (3.16 × 10^-7^)
20	rs1182531	57826397	34.96 (3.37 × 10^-9^)	33.67 (6.53 × 10^-9^)
21	rs1041778	22747323	22.72 (1.87 × 10^-6^)	23.25 (1.42 × 10^-6^)
22	rs713756	43118847	28.16 (1.12 × 10^-7^)	31.04 (2.53 × 10^-8^)

^a^Chr, chromosome. The values of statistic χ^2 ^and the related *p*-values are from program PLINK http://pngu.mgh.harvard.edu/~purcell/plink/anal.shtml.

## Discussion

The results of our genome-wide scan provided a large number of SNPs that have high test scores. One reason for this is the large sample size of NARAC data. For further study, one may start with the regions that contain the SNPs that have highest test scores, i.e., the regions with strongest signals. The Hotelling's *T*^2 ^tests do not adjust for population substructures. Thus, some of the strong signals could be due to false positives. Further study is necessary to clarify these issues.

The Hotelling's *T*^2 ^test does not include a multiplicity adjustment. However, we can perform a very conservative (assuming independence of the tests) Bonferroni analysis as follows. In the RA study, we analyzed 490,163 SNPs in total across the whole human genome. Therefore, there are 490,163 *T*_*H *_(or *T*_*G*_) tests. For the most significant SNP (rs2900180) with the highest test scores on chromosome 9 on the right hand-side of Table 1, the *p*-value of *T*_*H *_= 34.21 is 4.95 × 10^-9^. After adjusting for the multiple tests, the probability to get such a result by chance is 4.95 × 10^-9 ^* 490,163 = 0.0024. Hence, the result is still very significant. For the least significant SNP (rs10985073), the *p*-value of *T*_*H *_= 28.16 is 1.12 × 10^-7^. After adjusting for the multiple tests, the probability to get such a result by chance is 1.12 × 10^-7 ^* 490,163 = 0.055, which is close to the 0.05 significance level. The rest of the results in Tables 1 and 2 can be analyzed similarly.

We compared our results with those in literature [2-6] and found them to be consistent. In addition, we analyzed the data using PLINK and found similar results as those of Table 1 and Table 2; partial results are presented in Table 2. Hence, our results for analysis of data from candidate studies and genome-wide scans showed that the Hotelling's tests performed well. Furthermore, we could jointly use multiple SNPs in analysis as we did for data of chromosomes 6 and 9.

## Conclusion

We performed a genome-wide association scan for RA data by applying Hotelling's *T*^2 ^tests. In the candidate regions of the HLA-DRB1, TRAF1-C5, and PTPN22 genes, we identified SNPs that have the highest test scores across chromosomes 6, 9, and 1, respectively. Given the encouraging results in the candidate gene regions, the regions containing SNPs with high test scores are of interest for further investigation to map genes which have modest effects on RA. We provided the SNPs and their positions that had the largest scores for each chromosome. The regions of these SNPs deserve more investigation to map RA genes.

## List of sbbreviations used

GAW: Genetic Analysis Workshop; NARAC: North American Rheumatoid Arthritis Consortium; RA: Rheumatoid arthritis; SNP: Single-nucleotide polymorphism

## Competing interests

The authors declare that they have no competing interests.

## Authors' contributions

CIA and RF conceived the main idea of the study. LC, MZ, and WVC performed statistical analysis under the direction of CIA and RF. LC, CIA, and RF wrote the manuscript. MZ and WVC provided comments to improve the writings of the manuscript. All authors read and approved the final manuscript.
